# Research Translation to Promote Urban Health in Latin America: The SALURBAL Experience

**DOI:** 10.1007/s11524-024-00877-5

**Published:** 2024-06-27

**Authors:** S. Claire Slesinski, Katherine Indvik, Tonatiuh Barrientos-Gutierrez, Andrea Bolinaga, Waleska Teixeira Caiaffa, Francisco Diez-Canseco, J. Jaime Miranda, Daniel A. Rodriguez, Olga L. Sarmiento, José G. Siri, Alejandra Vives Vergara, Ana V. Diez Roux, The SALURBAL group

**Affiliations:** 1 Urban Health Collaborative, Drexel University Dornsife School of Public Health, Philadelphia, PA USA; 2 Center for Research in Population Health, National Institute of Public Health, Cuernavaca, Morelos Mexico; 3 Belo Horizonte Observatory for Urban Health, Belo Horizonte, Brazil; 4 Faculty of Medicine, Federal University of Minas Gerais, Belo Horizonte, Brazil; 5 CRONICAS Center of Excellence in Chronic Diseases, Universidad Peruana Cayetano Heredia, Lima, Peru; 6 Faculty of Medicine and Health, Sydney School of Public Health, University of Sydney, Sydney, Australia; 7 Institute of Transportation Studies, University of California, Berkeley, CA USA; 8 Department of City and Regional Planning and Institute Transportation Studies, University of California, Berkeley, CA USA; 9 Department of Epidemiology, University of Los Andes, Bogotá, Colombia; 10 Independent Consultant, Urban Health, Philadelphia, PA USA; 11 Departamento de Salud Pública, Escuela de Medicina, Pontificia Universidad Católica de Chile, Santiago, Chile; 12 Centro de Desarrollo Urbano Sustentable, CEDEUS, Pontificia Universidad Católica de Chile, Santiago, Chile

**Keywords:** Policy making, Policy engagement, Research translation, Urban health, Health equity, Cities, Latin America

## Abstract

In highly urbanized and unequal Latin America, urban health and health equity research are essential to effective policymaking. To ensure the application of relevant and context-specific evidence to efforts to reduce urban health inequities, urban health research in Latin America must incorporate strategic research translation efforts. Beginning in 2017, the Urban Health in Latin America (SALURBAL) project implemented policy-relevant research and engaged policymakers and the public to support the translation of research findings. Over 6 years, more than 200 researchers across eight countries contributed to SALURBAL’s interdisciplinary network. This network allowed SALURBAL to adapt research and engagement activities to local contexts and priorities, thereby maximizing the policy relevance of research findings and their application to promote policy action, inform urban interventions, and drive societal change. SALURBAL achieved significant visibility and credibility among academic and nonacademic urban health stakeholders, resulting in the development of evidence and tools to support urban policymakers, planners, and policy development processes across the region. These efforts and their outcomes reveal important lessons regarding maintaining flexibility and accounting for local context in research, ensuring that resources are dedicated to policy engagement and dissemination activities, and recognizing that assessing policy impact requires a nuanced understanding of complex policymaking processes. These reflections are relevant for promoting urban health and health equity research translation across the global south and worldwide. This paper presents SALURBAL’s strategy for dissemination and policy translation, highlights innovative initiatives and their outcomes, discusses lessons learned, and shares recommendations for future efforts to promote effective translation of research findings.

**Supplementary Information:**

The online version contains supplementary material available at 10.1007/s11524-024-00877-5.

## Background

Policy decisions impact almost every aspect of urban life: food choice, sleep quality, air quality, housing, recreation, social interaction, physical activity, safety, access to employment, and more [1, 2]. As a result, the health of urban residents and the distribution of health outcomes across residents are especially relevant domains for policymaking [3, 4]. This is especially true in Latin America where, in 2018, 81% of the region’s population resided in cities. Urbanization has occurred faster than in other regions, with 90% of the population expected to live in urban settings by 2050 [5]. This rapid urbanization process has taken place within a highly unequal environment. With a Gini coefficient of income of nearly 0.5, Latin America is the most unequal region globally, and a large portion of the region’s population lives in poverty [6]. These two factors have converged to create significant planning and environmental challenges with implications for health, including unmanaged and fragmented development [7, 8] urban sprawl [9], residential segregation by race and social class [10], a large proportion of the population living in inadequate shelter conditions [11], traffic delays with long commuting times [12], poor air quality [13], and violence [14, 15].

Latin American policymakers need timely access to context-specific and relevant evidence to inform actions to address these challenges, reduce inequities, and improve health in cities. Yet locally relevant evidence remains scarce due in part to low overall research output in the region, a byproduct of a lack of training opportunities and limited funding [16, 17]. Funders who do support health research in Latin America tend to be based elsewhere, with priorities that often do not align with those of policymakers in the region [18, 19]. Even where relevant research exists, researchers are rarely in a position to dedicate time and resources to effective dissemination, as funding seldom supports engagement with policy actors and communities [17]. Examples of effective dissemination are therefore scarce and poorly documented [20–22], and little is known about best practices for translating evidence to inform policy action in Latin America.

Even when evidence is effectively disseminated, policymakers may not make use of it. In 2011, a qualitative study in low- and middle-income countries revealed barriers to the uptake of health research for policy and practice, emphasizing limited technical capacity among policymakers to understand research, and diverse political influences such as competing interests, adverse incentives, resource and capacity deficits, inadequate institutional mechanisms, and rapid political turnover [23]. More than 10 years later, a gathering of Latin American academics and policy stakeholders generated a similar list, indicating that these challenges remain relevant and that region-wide solutions for translating urban health research into policy are still needed [4]. Improved understanding is required regarding how to effectively structure, disseminate, support, and publicize evidence to overcome existing challenges.

The Salud Urbana en América Latina (“Urban Health in Latin America”) (SALURBAL) project was developed in response to the region’s unique urban challenges, gaps in region-specific urban health evidence, and the need for effective dissemination of evidence to policy actors to improve urban health and reduce urban health inequities. The project, which emerged from the Urban Health Network for Latin America and the Caribbean, was launched in early 2017 and was implemented by a coalition of research institutions and non-governmental organizations coordinated by Drexel University’s Urban Health Collaborative. SALURBAL’s work was guided by four aims, one of which focused specifically on engaging with the scientific community, the public, and policymakers to disseminate findings and translate them into policies and interventions [24].

This paper presents a description of the implementation and outcomes of SALURBAL’s research translation activities. First, the project’s overall strategy and approach is described. Next, activities designed to engage regional and global policy actors are presented. This is followed by an overview of activities designed to engage local policy actors and decision-makers at the national and sub-national level. Finally, selected evidence of our reach and impact is presented, followed by a summary of lessons learned and recommendations for future efforts.

## SALURBAL’s Research Translation Strategy

Within the framework of SALURBAL’s aim to engage the scientific community, the public, and policymakers to disseminate findings and translate them into policies and interventions, the project developed four specific objectives:

1. Ensure the relevance of research to high-priority policy issues regionally and globally through the engagement of stakeholders in research and evaluation processes.
2. Disseminate findings broadly to the scientific community, the various publics, and policy actors.
3. Promote new ways of thinking among policy actors and other stakeholders about the drivers of urban health and the types of policies and interventions that could improve health and sustainability in cities.
4. Advocate for and support the translation of research findings into policies and interventions.

As the project entered its first months, the SALURBAL team developed a strategy outlining three categories of activities (Table 1) to support these objectives. While scientific publications and presentations are vital for sharing research findings, this paper focuses on translation efforts beyond traditional academic channels.

**Table 1:** SALURBAL's research translation strategy: policy engagement and dissemination activities

Category	Goal	Specific activities
1. Events, activities, and materials targeting regional and global policy actors	Engage in regular dialogue with regional and global policy actors to understand their needs and priorities and communicate research results through materials and channels accessible to these actors	Policy and data briefs
		Knowledge-to-Policy forum
		Community-based system dynamics workshops
		Research dissemination webinars
		Partnership and collaboration with regional and global policy actors
2. Engagement activities targeting local policy actors and communities	Leverage and support local country hub teams to engage with local policy actors, develop context-specific and appropriate research questions and evidence, and communicate research findings to local actors from multiple sectors	Engagement in policy evaluation studies
		Dissemination and engagement events for local policy actors
3. Media outreach, communications, and dissemination	Build awareness of project activities and results across a wide-reaching audience through digital, social, and mass media	Digital dissemination and social media
		Media releases and outreach to journalists

**Fig. 1:**
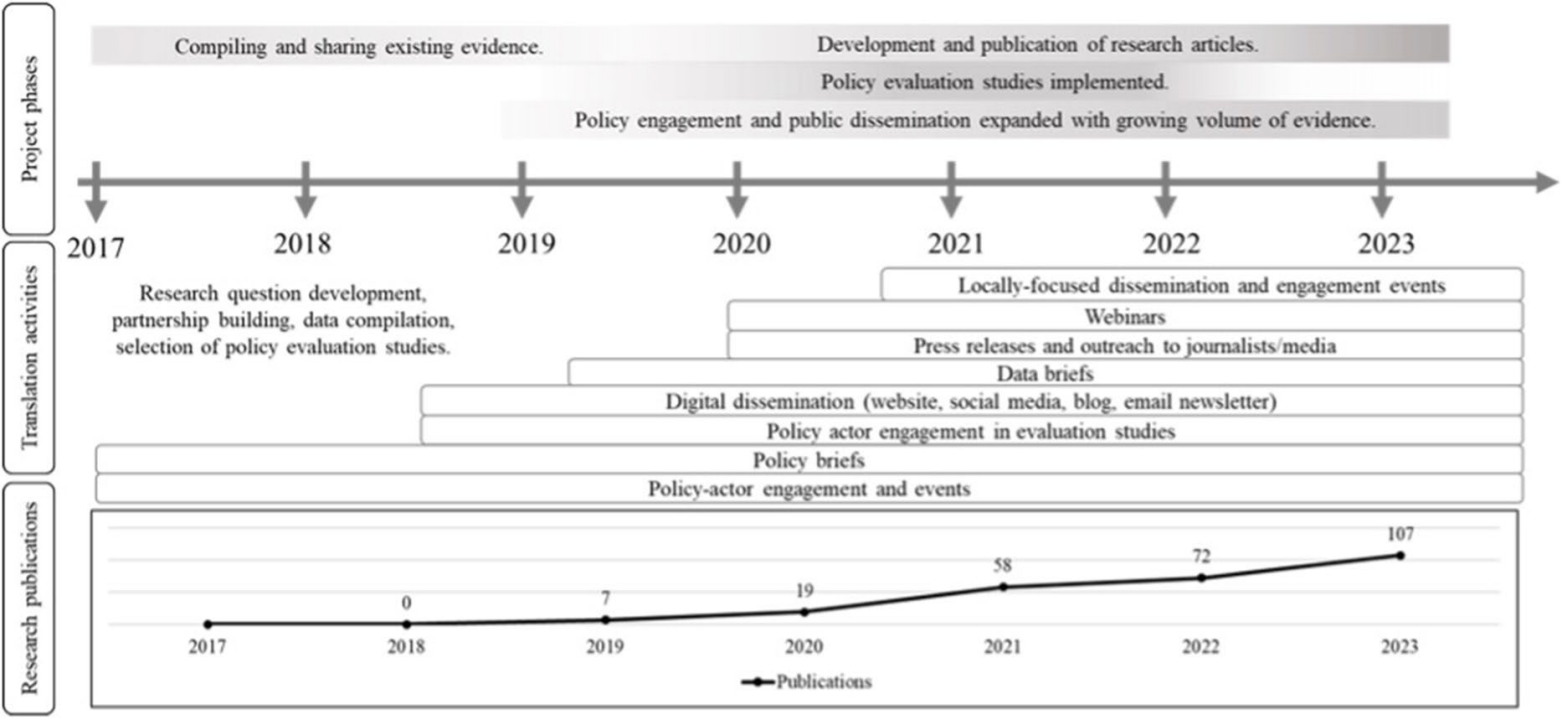
SALURBAL’s policy engagement and dissemination activities over time. Top: Project phases. Middle: New policy engagement and dissemination activities were initiated at each phase and continued throughout the entire project. Bottom: Cumulative number of peer-reviewed publications produced by the project through May 2023

SALURBAL’s research translation strategy was designed with flexibility to allow shifts over time in response to project activities and needs (Fig. 1). During the project’s first two and a half years (2017–2019), the research team focused on conducting literature reviews and compiling and harmonizing data [25]. During this time, policy engagement and dissemination efforts concentrated on strategy development, gathering and disseminating pre-existing evidence through policy briefs, identifying strategic collaborators, and building partnerships. Activities included networking and policy dialogue events, collaborative research design and systems mapping, and selection of SALURBAL’s six community-engaged policy evaluation studies (detailed below).

As the amount of evidence produced by SALURBAL increased during the third and fourth years of the project, the team incorporated additional policy engagement and dissemination activities, including press releases, data briefs, blog posts, an email newsletter, and webinars. During the project’s fourth year, the growing volume of evidence and results from policy evaluation studies warranted the implementation of locally targeted policy dialogue events. Over time, most existing activities were maintained as new activities were added, shifting to online platforms throughout the COVID-19 pandemic as needed.

Policy engagement and dissemination activities were implemented by team members across all partner institutions. This work was overseen by the Principal Investigator and the project’s Executive Committee with input and guidance from an external Advisory Panel, which included representatives from global, regional, and local policy actor organizations. At Drexel University, research translation work was coordinated and implemented by a Project Manager, a Communications Specialist, and a Policy Engagement Specialist. A Policy Working Group composed of country liaisons maintained relationships with local policy partners, implemented local in-person and virtual events, and guided local dissemination efforts.

Because SALURBAL was an international project focused on Latin American cities producing globally relevant results, many written materials were produced in English, Spanish, and Portuguese. Virtual events were sometimes implemented with simultaneous translation or re-recorded in a second language.

## Events and Materials Targeting Regional and Global Policy Actors

SALURBAL engaged with and disseminated research findings to regional and global policy actors through various products and activities. These included policy and data briefs, a “Knowledge-to-Policy” forum, community-based systems dynamics workshops, webinars, and collaborations with regional and global policy partners.

### Policy and Data Briefs

SALURBAL developed policy and data briefs for policy stakeholders in English, Spanish, and Portuguese. All policy briefs and most data briefs included concrete recommendations for policymakers and planning officials. Policy and data briefs were disseminated digitally through the project website and on social media and were distributed at in-person events and meetings.

**Fig. 2:**
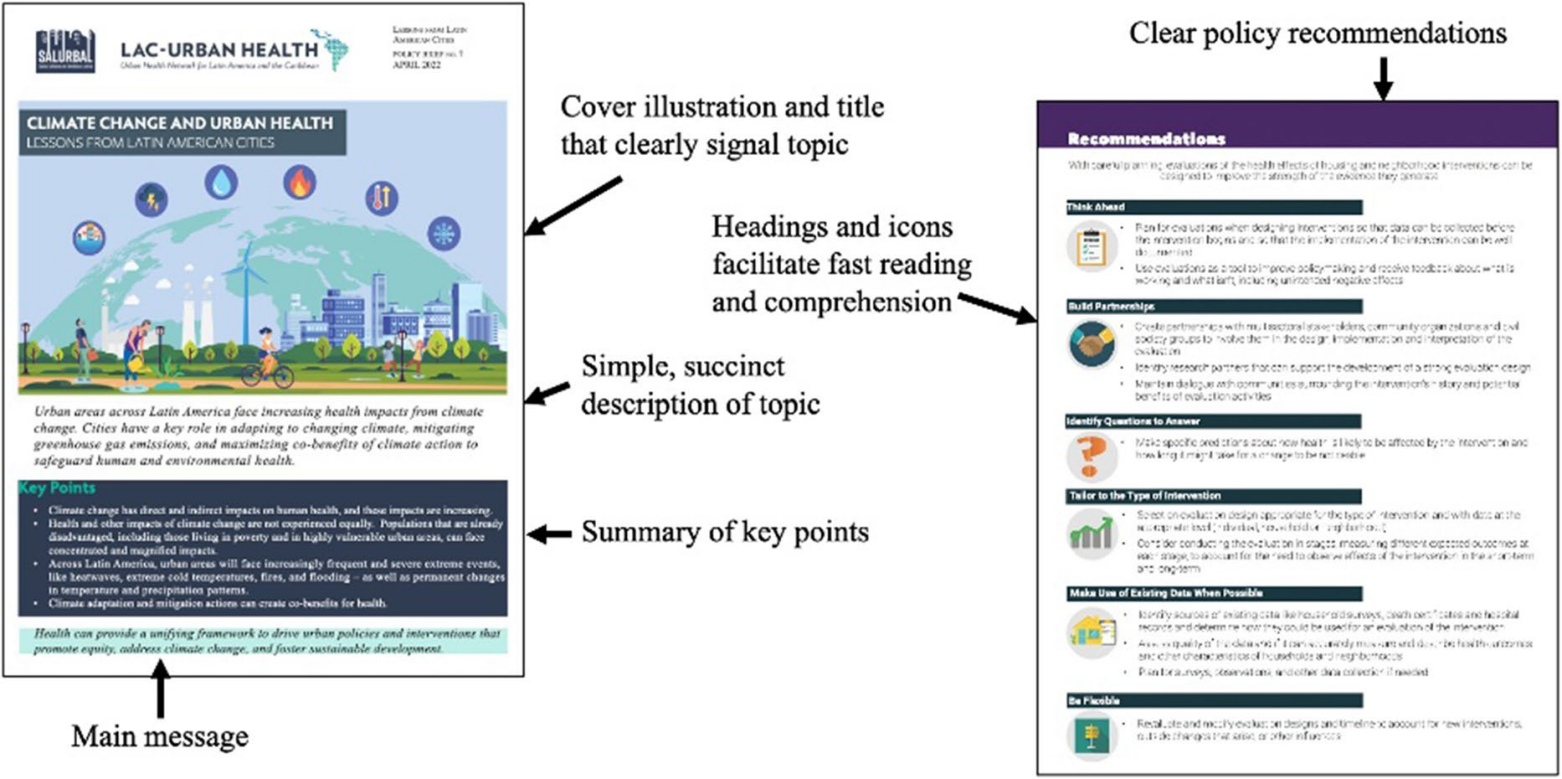
Highlighted sections from SALURBAL policy briefs. Design and content prioritize clarity, readability, and impact, incorporating visual elements (including icons) to support rapid interpretation and comprehension

SALURBAL’s policy briefs explored how policies across different urban sectors influence health and how such policies can be modified to improve health. Content included (1) examples of policies from across Latin America, (2) clear descriptions of links between these policies and health, and (3) recommendations. Figure 2 highlights selected sections from policy briefs.

**Fig. 3:**
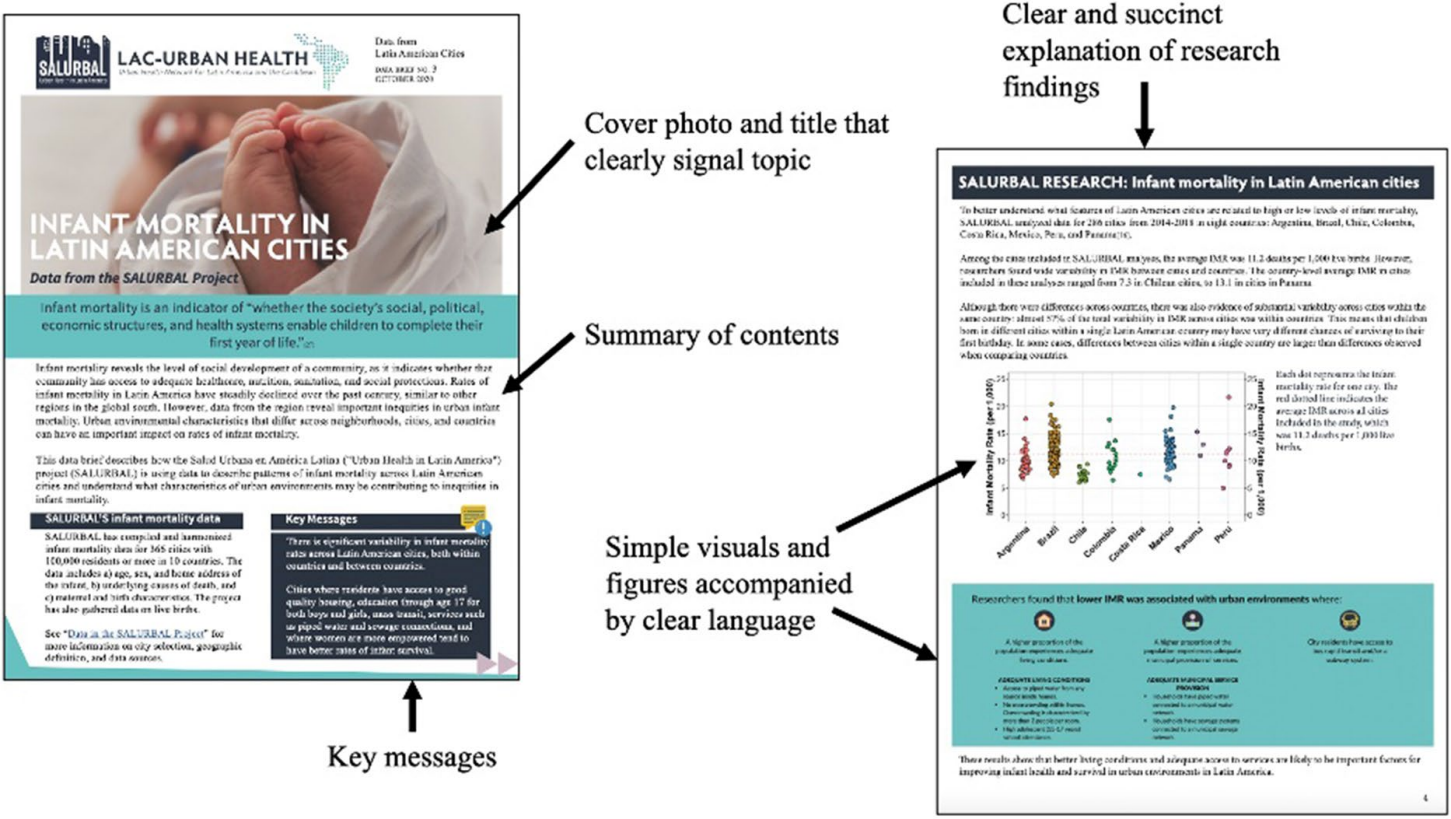
Examples from a SALURBAL data brief, highlighting features that facilitate understanding by non-scientists and policy actors, such as simple visuals and highlighted key messages

SALURBAL’s data briefs explored data and evidence for topics selected based on data needs in the region, emerging project results, and regional policy priorities. The briefs included (1) an overview of the health topic and how it connects to regional urban environments; (2) a summary of SALURBAL’s relevant data and research, including infographics; and (3) recommendations. Figure 3 highlights selected sections from data briefs.

A set of guidelines for brief development was informed by best practices in strategic communication and researchers’ experiences:

- Use simple language and minimize text.
- Use short, to-the-point sentences, which policymakers can copy and paste into their presentations.
- Focus on visually engaging content with a lot of color, graphics, and pictures.
- Create stand-alone visuals and infographics that can also be used on social media and in presentations.
- Feature logos from collaborating institutions, which policymakers can use to reinforce their arguments for urban policies that promote health.

In total, SALURBAL produced 15 briefs (Supplementary Table 1). Policy briefs covered topics across many urban sectors including transportation, housing, and climate change. Data briefs focused on mortality and life expectancy, COVID-19, and race and ethnicity data. SALURBAL’s briefs often represented opportunities for collaboration with strategic partners. Briefs were shared with local, regional, and global policy partners, at events, and through digital channels.

### Knowledge-to-Policy Forum

In 2018, SALURBAL hosted a “Knowledge-to-Policy forum” in Mexico City. This 2-day event engaged researchers, policymakers, representatives from regional and national non-governmental organizations, and representatives of civil society and regional and international cooperation. The goals of the event were to make regional stakeholders aware of SALURBAL, to connect these actors to the project in a formal way, and to solicit their input on how SALURBAL could best support policy processes to support urban health within the region.

At the time, SALURBAL was finalizing a first set of cross-sectional data [25] and preliminary descriptive analyses. Through a variety of participatory activities, the event (a) presented the project’s data and preliminary analyses while collecting feedback on how to increase policy relevance, (b) facilitated identification of options for translating research into urban policy, and (c) supported dialogue and exploration of opportunities for collaboration between SALURBAL and regional stakeholders.

Forum participants highlighted research topics and questions relevant to their work and identified opportunities for the regional scientific community to improve research translation. Participants expressed interest in a range of evidence and data related to many urban sectors including transportation, security, and governance. They also provided insight regarding data and research methods that could generate this evidence, including within-city and small area studies, policy and program evaluations, cost-benefit analyses, and the development of relevant frameworks and indicators. The event reaffirmed the enthusiasm of diverse international, regional, and national stakeholders for collaborating with SALURBAL to address the impacts of multiple sectors on urban health in Latin America. A report summarizing participants’ recommendations outlined proposed responses, which were integrated across the project’s work plans.

Although COVID-19 interrupted plans for a second in-person forum, SALURBAL continued to engage with many of the 2018 Forum participants, providing updates, receiving feedback, and contributing to numerous events organized by these actors and their institutions.

### Community-Based System Dynamics Workshops

One of SALURBAL’s research aims was to employ systems thinking and simulation models to evaluate links between urban systems and urban health, health equity, and environmental sustainability in order to identify promising policies and interventions. One way in which these models can be developed and strengthened is through participatory group modeling activities. These activities bring together representatives from multiple disciplines and sectors to develop a shared understanding of specific complex systems [26]. More than 60 policy stakeholders from across the region participated in three model building workshops during 2018 and 2019 in Peru, Guatemala, and Brazil [26]. Participants included researchers, policymakers, civil society, and private sector representatives.

Workshops explored links between urban transportation systems, urban food systems, health outcomes, and health inequities, with participants providing feedback on research questions and practical implications. Several participatory approaches were implemented [26]. The insights generated informed the selection of research questions for epidemiological studies and the development of agent-based models of Latin American food [27] and transport systems to assess the potential impacts of policy interventions. Published findings have contributed to the field of community-based system dynamics [26, 28, 29].

Beyond research-focused objectives, workshops also aimed to (a) put health and health equity on the agenda of policymakers potentially unaware of how their work influences health and (b) learn about and expand the mental models (i.e., cognitive representations of real dynamic systems) of diverse stakeholders surrounding transportation, food systems, and health.

### Global Research Dissemination Webinars

Beginning in 2020, the SALURBAL team hosted a series of virtual events to present project findings to policymakers, civil society representatives, community members, and other researchers across the region and globally. These live events were held in English and later recorded in Spanish. Each event featured two or three project researchers and one or more external partners discussing the potential applications of the results presented. For example, a November 2020 webinar on road safety presented findings from three SALURBAL studies and included two discussants representing important academic and non-academic global organizations. Nearly 400 people attended these three events, which are listed in Supplementary Table 2.

### Partnership and Collaboration with Regional and Global Policy Actors

SALURBAL’s approach to policy engagement included strategic outreach and partnership building with regional and global organizations. A description of SALURBAL’s partnerships is found in Table 2. The project’s multifaceted efforts to engage with policy actors through its advisory panel and additional efforts have allowed SALURBAL to become a resource for organizations seeking to deepen and strengthen their urban health activities in the region. Two prime examples of this are the Pan American Health Organization (PAHO) and the Inter-American Development Bank (IADB) (see details in Table 2).

**Table 2:** SALURBAL’s contributions to tools and materials that support urban decision-makers and planning professionals

Partner	Location/scale	Partnership activities
Pan American Health Organization (PAHO)	Regional partner	Participation in SALURBAL advisory panel; co-development of a SALURBAL policy brief, “Health in All Urban Policies”; collaboration on the development of PAHO-coordinated Healthy Municipalities, Cities, and Communities criteria; dissemination of SALURBAL research and data at a Regional Meeting of Mayors for Healthy Municipalities, Cities, and Communities in the Region of the Americas
World Health Organization (WHO)	Regional/global partner	Participation in SALURBAL advisory panel; contributions to the Spanish language version of the WHO Sourcebook directory: Integrating health in urban and territorial planning
Inter-American Development Bank (IADB)	Regional partner	Participation in SALURBAL advisory panel; SALURBAL-led development of framework, tools, and piloting for IADB’s integration of health into housing and urban redevelopment intervention evaluations (led by *Universidad de los Andes*); SALURBAL participation in development of IADB video, “Health and city: Your zipcode matters more than your genetic code” [30]
CAF (Development Bank of Latin America)	Regional partner	Participation in SALURBAL advisory panel; SALURBAL contribution to the development and dissemination of the CAF “Guía para Ciudades Más Saludables” (Guide for Healthier Cities)
CDP (formerly the Carbon Disclosure Project)	Regional/global partner	Participation on SALURBAL advisory panel; consultation on integration of health within CDP Cities Questionnaire; participation in SALURBAL ancillary study on the health impacts of climate change–related extreme temperatures in Latin American cities; co-development of two briefs on greenspace, water security, heat, and health in cities
C40	Regional/global partner	Participation on SALURBAL advisory panel; collaboration on special policy brief: “Promoting urban health equity in a post-COVID world: A view from Latin America”

Over the course of the project, PAHO’s Department of Social and Environmental Determinants for Health Equity emerged as a key SALURBAL partner. Grounded in shared goals surrounding health equity in Latin American cities, PAHO representatives and SALURBAL team members began meeting regularly in 2018. This ongoing collaboration was eventually formalized with a Memorandum of Understanding and has fomented numerous opportunities for each organization to support, inform, and increase visibility of the other’s work (as described in Table 2).

Another important partner emerged in 2020, when representatives from the Housing and Urban Development Division of the IADB contacted SALURBAL. Seeking to integrate a health focus within their work, the Division reached out to SALURBAL after reviewing the project’s policy brief, “Planning health evaluations of housing and neighborhood interventions.” Several consultations evolved into a partnership between IADB and SALURBAL’s team at *Universidad de los Andes* (Colombia), culminating in the development of a framework and evaluation toolkit for integrating health into IADB’s housing and urban development work, and pilot testing of the toolkit in Colombia and Chile by the SALURBAL teams in each country. SALURBAL findings were subsequently featured in an IADB documentary highlighting the influence of neighborhood environments on urban health and health equity in Latin America [30].

## Engagement Activities Targeting Local Policy Actors and Communities

SALURBAL engaged with and disseminated research findings to local policy actors and communities through six policy evaluation projects and through locally focused dissemination efforts and events.

### Policy Evaluation Projects

In early 2018, SALURBAL requested proposals for innovative studies evaluating the health and environmental impacts of urban policy interventions in Latin America that fell within four broad categories: mobility and emissions control, comprehensive urban development, social inclusion and reduction of social inequalities, and the promotion of healthy behaviors. Six projects were selected and implemented in close partnership with city governments and communities in Mexico, Colombia, Peru, Chile, and Brazil. The policies and interventions evaluated involved many aspects of the urban environment: road safety, active transportation, mass transit, food environments, housing quality, and urban renewal. Descriptions of these studies and their community and policy engagement activities are presented in Table 3.

**Table 3:** Descriptions of SALURBAL’s policy evaluation projects, including details of the policies evaluated, outcomes measured, and engagement with local partners

Description of policy or intervention evaluated	Health and environmental outcomes measured	Community and policy engagement
Urban Regeneration, Quality of Life, and Health (RUCAS) | *Viña del Mar and Santiago, Chile* [31]		
Participatory intervention upgrading existing social housing units and constructing new units, installing community green spaces and recreation facilities, repaving streets and sidewalks, and improving lighting of streets and parks, among others	Self-rated health, respiratory conditions, mental health, and housing satisfaction	Chilean Ministry of Housing and Urbanism (MINVU), researchers from the Center for Sustainable Development (CEDEUS), and community leaders from the two communities participating in the study. More than 20 dissemination and dialogue events with community leaders, MINVU representatives and other researchers. Public-facing website: www.estudioRUCAS.cl
Belo Horizonte Vila Viva Project (BH-Vila Viva) | *Belo Horizonte, Brazil* [32]		
Improvements in sanitation, housing conditions, neighborhood streets, and the provision of leisure and recreational spaces and resources in informal settlements; initiatives to enhance the quality of life including economic empowerment through microfinance, and establishment of legal ownership over property by providing land titles	Deaths, asthma rates, mosquito-borne disease, and risk factors related to non-communicable diseases	Urbanization and Housing Company of Belo Horizonte (URBEL), the organization implementing the Vila Viva program. Data collection was made possible through the generous cooperation and participation of residents of the Serra slum and Cabana slum of Belo Horizonte
Urban transformations and health: The case of TransMiCable in Bogotá (TrUST) | *Ciudad Bolívar, Bogotá, Colombia* [33]		
Installation of a new cable car transit system (TransMiCable), physical improvements to homes, geomorphological hazard reduction, addition of tourist office, local markets, community centers, citizen services office, and recreational and cultural infrastructure	Inhaled air pollution, physical environment perceptions; access to recreational and cultural facilities; transport accessibility; employment; social capital; leisure time; leisure and transport-related physical activity; health-related quality of life; respiratory diseases; homicides	The conceptual framework and research plans for this study were designed and validated through numerous consultations with diverse actors throughout the study, including representatives from academia; public sector representatives including officials from government institutions and the managing and coordinating entity of the public transportation system of Bogotá; and civil society, including two community leaders from Ciudad Bolívar and community members. Public-facing website: https://fabiancpl.github.io/transmicable/
Evaluation of new road traffic regulations, specifically speed limits, on crashes, fatalities, and air pollution in Mexico City | *Mexico City, Mexico* [34]		
Implementation of new traffic regulations in 2015 (including stricter speed limits, monitoring, and enforcement including speed cameras and monetary fines) and subsequent changes to these regulations, notably removal of monetary fines for speeding, in 2019	Collision rates, rates of collisions with injuries, mortality trends, and air pollution (NO_2_ and PM_2.5_)	Collaboration and engagement with the National Autonomous University of Mexico (UNAM), AXA insurance, the former head of traffic engineering for Mexico City, representatives of government institutions including the National Commission for Accident Prevention (CONAPRA), and non-governmental organizations involved in road safety including Céntrico, the Mexican Association of Insurance Institutions (AMIS), the Institute for Transportation and Development Policy (ITDP), and public servants at the city’s mobility department
Effect of new bicycling infrastructure on urban health: A natural experiment in Mexico City | *Mexico City, Mexico*		
EcoBici bike share system and expansion of cycling infrastructure in response to the COVID-19 pandemic	Changes in bicycle ridership and transit mode usage; transport-related physical activity; variations in EcoBici user demographics; contribution of EcoBici in meeting physical activity recommendations; neighborhood characteristics associated with EcoBici use	Engagement and collaboration with the Mexico City Secretariat of Mobility (SEMOVI). Data from the city-run EcoBici program received directly from SEMOVI
Evaluating the implementation and effects of warning advertising on food labels in Peru: A mixed-methods study | *Lima, Peru* [35–37]		
Law for the Promotion of Healthy Eating for Children and Adolescents: Octagon-shaped nutrition warnings required to be placed on the packaging of processed foods and beverages containing trans-fat or classified as “high” in sodium, sugar, or saturated fats	Adolescents’ food choices; implementation of food labeling changes by industry; and changes to ingredients (reformulation) or marketing by industry in response to the law’s implementation	Close collaboration with local administrators from Schools Fe y Alegría N° 3 and N° 37

### Dissemination and Engagement Events for Local Policy Actors

SALURBAL applied two approaches to local outreach and dissemination. First, SALURBAL team meetings, held throughout the region, were accompanied by public events engaging local practitioners and policymakers. These events leveraged country teams and partner institutions to build project visibility, connect with local stakeholders, and highlight project results with relevance to local priorities. Second, SALURBAL developed the *Diálogos SALURBAL* series, led by local team members and designed to present policy-relevant research results to local policymakers and community members. *Diálogos* were held virtually during the COVID-19 pandemic, with in-person events resuming in 2023. Each event was adapted to local contexts and audiences, leveraging the strength and connections of SALURBAL country-based teams and dissemination support from the central project team. *Diálogos* were conducted in local languages and/or with simultaneous translation. An overview of SALURBAL’s locally focused dissemination and engagement events is found in Supplementary Table 3.

One unique *Diálogos* SALURBAL event, “Urban transformations, community participation, and health: Lessons from Brazil, Chile, and Colombia,” was co-hosted by three of SALURBAL’s country hubs [38]. Research presentations from each evaluation were followed by commentary and discussion among community members directly impacted by the interventions. Policy actors and decision-makers then reflected on the of the urban transformation projects. The event was held virtually and included simultaneous translation between Spanish and Portuguese. The discussions during this event allowed for collective interpretation of preliminary study findings, and an assessment of lessons learned during the implementation of both the intervention itself and the evaluation.

## Media Outreach, Communication, and Dissemination

SALURBAL disseminated its research through digital communications and news media using a website, a digital newsletter, social media, press releases, and direct engagement with journalists. A social media and communications strategy defined target audiences for SALURBAL’s research findings and outlined an approach for connecting with each type of audience member. For example, researchers and representatives from international non-governmental organizations may be more easily reached through Twitter and an email newsletter, while local and national policy actors may prefer to receive information through policy briefs and events.

Dissemination of each project publication was informed by each set of findings. For example, publications containing evidence more directly relevant to the public and for policy- and decision-making were distributed through media releases and social media campaigns, while publications focused on methods or communicating complex results most relevant to scientists and academics were disseminated through SALURBlog posts. All original research papers developed by SALURBAL were published under Wellcome Trust’s open access policy, uploaded to the online publications repository, and shared via Twitter.

SALURBAL’s digital dissemination channels included a website, an email newsletter, a blog (“SALURBlog”), and social media. A description of the information disseminated through each of these platforms is found in Table 4.

**Table 4:** Descriptions of SALURBAL’s digital dissemination channels and content

Channel	Description
Website	A hub for all information related to the project, including all SALURBAL publications, descriptions of activities, policy and data briefs, information about upcoming events, recordings, and slides from past events, the SALURBlog, and media releases and other news
Email newsletter	A quarterly digest of updates from SALURBAL and other ongoing urban health initiatives in the Latin America region, including events, publications, policy and data briefs, and media releases and news
SALURBlog	An online platform for regional and global dialogue and discussion on urban health in Latin America. Contributors, including SALURBAL researchers, are experts in urban health, urban planning, and urban policy from across the region. Blog posts include thought pieces and commentaries on recent events or trending topics, and explanations of SALURBAL research that integrate a policy discussion and policy recommendations
Social media	SALURBAL social media accounts on Twitter, Facebook, Instagram, LinkedIn, and YouTube. Twitter provides an opportunity to reach a large audience with project updates and simplified explanations of research findings using threads and by linking to blog posts, data briefs, and more. Instagram is ideal for sharing visual media such as simple infographics. LinkedIn connects SALURBAL to professional networks, organizations, and initiatives. YouTube is a platform for sharing recordings of webinars, virtual events, and other videos

SALURBAL developed media releases for select publications that aligned with regional policy priorities and had especially significant results. Occasionally, press releases were tailored for specific cities or countries. Media releases were translated into Spanish, Portuguese, or both.

Once finalized, media releases were added to a journalist-targeted website, which included all media releases, author biographies and photos, and a media kit. The SALURBAL Communications Specialist shared media releases with media contacts at regional and international outlets. Policy Working Group members throughout the region shared media releases with local outlets. In some cases, news articles derived from these media releases included interviews with one or more authors.

## Reach and Impact of Research Translation Activities

SALURBAL made efforts to track metrics and capture case studies related to its reach and impact beyond the research sector. The project’s reach through digital dissemination can be summarized using metrics on views, visits, and subscribers. Impact on policy and civil society is captured through case studies.

While the “real-world” impact of SALURBAL’s communications and dissemination activities is difficult to quantify, the broad reach of the project’s messages is clear, especially in terms of website visitors (more than 83,000 unique webpage visits from 170 different countries) and average monthly Twitter impressions^1^ (approximately 16,000 impressions per month on average over the lifespan of the project). The quarterly email newsletter reached more than 470 subscribers, and the project’s videos have more than 2700 unique views on YouTube. More detail on these indicators can be found in Supplementary Table 4. SALURBAL research has also been highlighted in high profile media outlets such as El País (Spain), CNN (International), and BBC Mundo (International) along with national news outlets in the region like *El Espectador* (Colombia), *La Tercera* (Chile), and *La Nación* (Argentina).

Team members have identified evidence of SALURBAL’s impact on policymakers and policy processes through its translation activities. For example, qualitative research conducted with participants who attended a systems workshop in São Paulo, Brazil, found that the workshop influenced how they thought about urban policy and promoted systems thinking [39]. Participants reported that the workshop allowed them to “construct a shared language and understanding of urban challenges” and to improve their understanding of how food and transportation systems are interconnected. One year after the workshop, participants also reported increased capacity for applying systems thinking approaches within their own work and noted that their understanding of healthy urban environments had expanded significantly. Regarding the impact of SALURBAL’s policy and data briefs, correspondence from an Inter-American Development Bank Housing and Urban Redevelopment Division representative expressed that SALURBAL stood out as a useful resource, with “briefs that illustrate in a very visual and simple way the health outcomes of urban inequality … and provide the ground for evaluating health outcomes of urban policies” [40].

SALURBAL also directly contributed expertise and scientific guidance to various policy processes that were systematically tracked as case studies. Team members’ engagement in policy processes has included advocacy related to specific laws, informing government officials of available evidence as they design interventions, presenting policy evaluation results, participating in the development of strategic plans, and identifying and recognizing urban initiatives with relevance to urban health. These case studies are presented in Table 5.

**Table 5:** Highlighted contributions of SALURBAL to policy development and implementation processes

Policy or initiative	Location/scale/SALURBAL partner	Description
Bill 5504 (*Ley de Promoción de Alimentación Saludable*)	SALURBAL team; Guatemala	INCAP Guatemala consultation and advocacy for Bill 5504 (*Ley de Promoción de Alimentación Saludable*, law for the promotion of healthy eating)
COVID-19 pandemic response	SALURBAL team; Mexico City, Mexico	Collaboration with Mexico City government to inform efforts to reduce contagion on mass transportation systems during the pandemic
Food label law	Food label policy evaluation team; Lima, Peru	Presentation of Lima food labels evaluation results to Peruvian Ministry of Education contributed to their understanding of the success and impacts of the implementation of this law
Urban Bogotá 2022-2035 Plan	TrUST policy evaluation team; Bogotá, Colombia	Presentation of TransMiCable evaluation results to Planning and Mobility public officials, informing the development of similar interventions, such as the next cable car to be implemented in the evaluation’s control group area and will be inaugurated in 2025. This was integrated into the Urban Bogotá 2022–2035 plan
Housing renovation (Equipo de Renovación Urbana of the Ministerio de Vivienda y Urbanismo (MINVU) and SEREMI V Región.	RUCAS policy evaluation team; Viña del Mar and Santiago, Chile	Sharing of evaluation results with policy implementers has led to the introduction of improvements to the design of the dwelling intervention in one of the study sites. Study results have been requested by implementers as an input for their own design processes. Housing renovation implementers also contributed with ideas for additional evaluation analyses, the results of which served to highlight and/or confirm the heterogeneity in the distribution of habitability problems. Community leaders have used RUCAS results to back their demands concerning dwelling-related health problems
Co-development of the *Premio Ciudad* in Peru	Lima Cómo Vamos, Lima, Peru	SALURBAL participation in development of prize and selection of winners. The annual prize recognizes Peruvian municipalities implementing projects, best practices, and demonstrating leadership that contributes to urban health and wellbeing. Prizes are awarded to municipal governments, civil society, and citizen groups or movements

## Challenges and Lessons Learned

Below, we summarize key challenges and lessons learned during more than 6 years of SALURBAL’s dissemination and policy engagement efforts to promote urban health.

### Adapting to the Policy Context

#### Urban Health Needs a Paradigm Shift

Many regional and global policy actors are unaware of the “health in all policies” framework [3] and do not consider potential health impacts and opportunities when developing policies and interventions related to urban planning, transportation, food systems, housing, and the environment, as our team observed during many conversations with multisector stakeholders. Urban health researchers must contribute to a broader conversation about the influence of urban systems on health and health equity, shifting away from a sole focus on healthcare provision toward evidence-based policy that promotes health across all urban sectors.

#### Researchers—and Research—Need the Flexibility to Take Advantage of Opportunities as They Arise

Policy windows often open quickly and unexpectedly, and researchers must be ready to take advantage of opportunities as they come and pivot as necessary. SALURBAL had the flexibility to strategically fund policy evaluation studies as opportunities emerged in the region, including opportunities that emerged as a result of the COVID-19 pandemic [41, 42]. We also ensured an openness to engage with partners as we were approached and to share data aligned with their needs. The infrastructure support available to the project and the flexibility to reorient as needed was critical to our success. This type of flexibility is often lacking in research funding; in the case of SALURBAL, it resulted in part from establishing research dissemination and policy impact as priorities from the project’s outset.

#### Researchers Need Training in Policy Outreach and Dissemination, and Policy Actors Need Training in Research

Academic education often neglects the capacity to communicate research findings with non-scientists and non-experts. Urban health researchers must be able to communicate effectively with the public, journalists, and policy actors to ensure that their policy-relevant research can be used to institute and implement healthier urban policies and interventions. Likewise, non-scientists working in urban policy and planning would benefit from a deeper understanding of what research can and cannot do. For example, improved understanding of the research process, appropriate interpretations of causality, and the significance of uncertainty inherent to many research results would enable urban policy actors to better collaborate with researchers. Though providing this kind of training is difficult and is rarely prioritized, SALURBAL was able to dedicate resources to both capacity building among our researchers and to creating spaces and materials to increase policy actors’ access to and understanding of project findings.

#### Clear and Simple Communication Is Vital

While dissemination through formal academic channels requires precise technical terminology and a focus on methods, dissemination to policy actors and the public requires simple language and a focus on the interpretation of results as a basis for action. Creating clear and simple communication that is also scientifically accurate and does not overstate or gloss over evidence gaps is difficult and requires staff trained in both public health research and communications. SALURBAL’s data and policy briefs were developed with input from communications and policy professionals, local researchers, and policy representatives in an effort to achieve this balance.

#### There Is Demand for Descriptive Data

Over the course of the project, exchanges with policy actors have repeatedly highlighted an interest in basic descriptive data, analyses, and maps. In Latin America, capacities for simple data analysis and presentation can sometimes be weak or lacking, especially in small- and medium-sized cities. A straightforward presentation of data such as life expectancy calculations for urban municipalities can reveal stark inequities that trigger societal pressure and political will for change. Funders need to be aware of the value of this information, as well as the many scientific challenges involved in generating and communicating it.

### Research Funding, Design, and Implementation

#### Explicitly Dedicate Funding and Human Resources to Policy Engagement and Dissemination Throughout the Life Cycle of Policy-Relevant Research Projects

SALURBAL’s mandate for research translation is unusual. The project’s funding from the Wellcome Trust’s *Our Planet Our Health* program included a focus on developing “a stronger evidence base about the impact of humans on ecosystems that will enable individuals and governments to make informed decisions to safeguard the health of the population and the planet” [43]. Accordingly, the project budgeted for and implemented engagement and dissemination from the start, not only after research had been conducted. Funding agencies and projects aiming to produce meaningful policy impact need to consider the time, effort, and investment required to cultivate policy partnerships and implement successful dissemination and engagement activities.

#### Let Local Researchers Guide Dissemination and Engagement Activities

SALURBAL’s dissemination and policy engagement activities were guided by the project’s Executive Committee, composed of researchers in Mexico, Colombia, Chile, Peru, Brazil, and the United States. Activities were designed and implemented by a Policy Working Group comprised of representatives across partner institutions in eight countries. This group leveraged existing resources and connections to support dissemination and engagement within local contexts throughout the region, which would have been difficult or impossible if implemented solely by the lead institution. Changing political circumstances create challenges for engagement, especially in highly polarized contexts, and only local experts can effectively navigate these complex circumstances.

#### Engage Policy Actors from a Wide Range of Sectors from the Beginning and Throughout the Project

Diverse partners contributed to SALURBAL’s development, including two United Nations institutions [24]. The incipient project also established an advisory panel including regional policy stakeholders from regional and international development agencies (including CAF, World Bank Group, and IADB), non-governmental organizations (including World Resources Institute, the International Society for Urban Health, and Lima Como Vamos), PAHO, and the WHO. Researchers and advisory panel members represent a range of sectors including urban planning and mobility, health systems, air quality and climate, social programs, and academia. The project engaged community members in research through its policy evaluation projects. The project’s *Diálogos SALURBAL* events engaged local policy actors and other stakeholders in the interpretation and dissemination of findings. Although these events created competing time demands and occasionally highlighted stark differences in opinions and priorities, the inclusion of these voices increased the relevance, reach, and applicability of SALURBAL’s research.

#### Policy Translation Research for the Latin American Region Remains Lacking

Evidence regarding how and why policymakers take up and apply urban health research remains sparse, especially in the global south. SALURBAL’s engagement with local policy and community actors highlighted a number of specific gaps. Our team developed and adapted a set of best practices for stakeholder engagement, but formal research is required regarding the most impactful strategies for presenting and leveraging research findings to inform policy processes. This research should address the complex institutional and information systems that influence knowledge translation, as well as key factors for sustaining long-term relationships between researchers and decision-makers.

#### Policy Change Is a Long-Term Effort and Is Influenced by Many Factors

Policy processes are rarely short or linear, and significant change is unlikely to occur within typical research funding periods. Moreover, documenting the impact of research during the intermediate stages of the policy process can be extremely challenging, and linking a specific policy change to a given study or initiative is difficult. Funders should have realistic expectations about how and when the policy process can be impacted, and researchers should take a broad approach to evidence translation, acknowledging the value of long-term, gradual shifts in world views and paradigms [44]. Recognizing the role of additional contextual factors is fundamental to identifying windows of opportunity, appropriately adjusting efforts and activities, and ultimately leveraging scientific evidence for policy change.

## Conclusion

The SALURBAL experience demonstrates that a multi-country collaborative effort to generate and disseminate evidence for action is possible but requires designated infrastructure, sustained support, and flexibility. SALURBAL explored a variety of stakeholder engagement and dissemination strategies to support the translation of multiple types of urban health research findings and evidence. Although it is possible to document policy impact in selected circumstances, linking SALURBAL actions to specific policy changes was difficult within the period of the project. Long-term, sustained efforts that are multifaceted and engage diverse policy actors in different ways are needed. Moreover, changes in public perceptions about the drivers of health and the actions needed to support health are fundamental to creating meaningful change. The need for political changes and ideological shifts must also be recognized and addressed. Despite these challenges, solid multi-country research partnerships that leverage lessons across a region, that promote engagement between policymakers and scientists, and that are sensitive to political opportunities can support meaningful policy change.

## Supplementary Information

Below is the link to the electronic supplementary material.

Supplementary file1 (PDF 227 KB)

## Footnotes

1 The number of times a tweet is included in the timeline of Twitter users.
