# Supplementary material for: Research Translation to Promote Urban Health in Latin America: The SALURBAL Experience
Source: J Urban Health. 2024 Jun 27;101(6):1069–86. doi: 10.1007/s11524-024-00877-5 (PMC11652544; doi:10.1007/s11524-024-00877-5)
Supplement: Supplementary file 1 — Supplementary file1 (PDF 227 KB) [file 11524_2024_877_MOESM1_ESM.pdf]

Supplement

Research translation to promote urban health in Latin America: the SALURBAL experience

Supplementary Table 1

Type	Year	Title	Notes
Policy	2017	Sustainable transport and urban health	Developed with input from a policy dialogue event in Bogotá, Colombia, in May 2017
Policy	2018	Food and urban health	Developed with input from the Economic Commission for Latin America and the Caribbean (ECLAC) and the United Nations University (UNU)
Data	2018	Data in SALURBAL	General overview of SALURBAL's compiled data
Policy	2019	Planning health evaluations of housing and neighborhood interventions	Features three of SALURBAL's policy evaluation projects, including notes on collaboration with policy partners.
Policy	2019	Health in all urban policies	Developed in partnership with the Pan American Health Organization (PAHO) and disseminated at the Third Regional Meeting of Mayors for Healthy Municipalities, Cities, and Communities in the Region of the Americas
Data	2019	Mortality and life expectancy in Latin American cities	Includes recommendations for local authorities regarding the completeness and quality of birth and death registrations; disseminated during a SALURBAL webinar on inequality in mortality outcomes in Latin American cities.
Policy	2020	Knowledge to policy translation for urban health	Developed with input from SALURBAL's Knowledge-to-Policy Forum
Policy	2020	Public transportation and active transportation during the pandemic	Informed by literature review requested by Mexico City government [26]
Data	2020	Infant mortality in Latin American cities	Features research findings regarding the influence of housing, education, transportation, sanitation, and women's empowerment on infant survival; disseminated during a SALURBAL webinar on inequality in mortality outcomes in Latin American cities.
Results	2020	Urban transformations and health: Results from the TransMiCable evaluation	Developed in partnership with the Ministry of Health of Colombia and the Planning department of Bogotá
Policy	2021	Green space and water security: Examples of climate change adaptation from the public and private sectors in Latin America	Developed in partnership with CDP

Data	2021	COVID-19 and urban health in Latin America and the Caribbean	Developed with data from SALURBAL's COVID-19 data dashboard that combines and visualizes open data from 11 country governments
Policy	2022	Climate change and urban health	Includes recommendations for both urban policymakers and public health researchers
Results	2022	Urban Transformations and health: Results from the evaluation of TransMiCable during the COVID-19 pandemic	Developed in partnership with the Ministry of Health of Colombia and the Planning department of Bogotá
Data	2023	Making the Invisible, Visible: Race, Racism and Health Data in Latin American Cities	Developed in partnership with the Ubuntu Center on Racism, Global Movements and Population Health Equity. Outlines barriers to and recommendations for advancing research and action on racial and ethnic health inequities in Latin America.

Supplementary Table 1: SALURBAL's policy and data briefs with notes on their content, development, and dissemination.

Supplementary Table 2

**Date**	**Theme/topic**	**Language(s)**
1 July 2020	Inequality in mortality outcomes in Latin American cities	English
4 August 2020	*Desigualdad en resultados de mortalidad en ciudades de América Latina*	Spanish
9 September 2020	Air pollution and health in Latin American cities	English
15 October 2020	*Contaminación del aire y salud en ciudades latinoamericanas*	Spanish
17 November 2020	Road safety in Latin American cities	English
1 December 2020	*Seguridad vial en ciudades Latinoamericanas*	Spanish

Supplementary Table 2: SALURBAL's research dissemination webinars are listed, noting the date each occurred or was released, the title or topic of the event, and the language.

Supplementary Table 3

**Date and location**	**Theme/topic**	**Host institution and local partners/ panelists**	**Language(s)**
19 May 2017 *Bogotá, Colombia*	Expert panels on sustainable transportation and health / *Panel de Expertos en Transporte Sostenible y Salud*	Universidad de los Andes<br>Bogotá legislative chamber<br>Organización Despacio<br>Bogotá health secretariat<br>World Resources Institute<br>Federal University of Minas Gerais	Spanish
15 November 2017 *Lima, Peru*	Local interventions and policies to improve urban health in Lima / *Intervenciones y políticas locales para mejorar la salud urbana en Lima*	Universidad Peruana Cayetano Heredia<br>Lima Como Vamos<br>German international development agency (GIZ)<br>Juegos Panamericanos	Spanish
18 May 2018 *Antigua, Guatemala*	Research and policy symposium on Urban Health Policies and	Institute of Nutrition of Central America and Panama (INCAP)	Spanish

	Interventions in Central America	Central American Integration System (SICA)<br>SALURBAL team<br>Local community, policy, and academic representatives	
24 May 2019<br>*Belo Horizonte, Brazil*	Health, Housing, and Neighborhood Interventions in Brazil / *Saúde, habitação e políticas urbanas no Brasil*	Federal University of Minas Gerais, Brazil<br>Local communities, policy audiences, technical experts, and researchers	Portuguese, English
24 October 2019<br>*Lima, Peru*	"Chelas Urbanas" series organized by the Lima Cómo Vamos Observatory	Lima Cómo Vamos<br>Universidad Peruana Cayetano Heredia (UPCH)<br>SALURBAL Executive Committee members and local road safety experts and urban stakeholders	Spanish
20 April 2020<br>*Virtual (Costa Rica)*	Webinar on Social distancing and inequality in Latin America	University of Costa Rica<br>SALURBAL researchers and Executive Committee members	Spanish
11 March 2021<br>*Virtual (Guatemala)*	*Obesidad y diabetes: Entendiendo el rol de nuestras ciudades* (Obesity and diabetes: Understanding the role of our cities)	INCAP, Guatemala<br>Local policymakers and urban planning officials	Spanish
5-6 May 2021<br>*Virtual (Brazil)*	*Equidade e Saúde Urbana no Brasil / Saúde e sustentabilidade: Desafios de política pública na cidade de Salvador* (Equity and urban health in Brazil / Health and sustainability: Public policy challenges in Salvador)	Center for Data and Knowledge Integration for Health (CIDACS), Brazil<br>Local officials<br>Local community members<br>SALURBAL researchers	Portuguese
27 May 2021<br>*Virtual (Chile)*	*Ciudad y Vivienda: Determinantes clave para la salud poblacional* (Cities and housing: Key determinants of population health)	Pontifical Catholic University of Chile (PUC)<br>University of Chile (UC)<br>Chilean Ministry of Housing<br>Local authorities	Spanish
2 September 2021<br>*Virtual (Brazil, Chile, Colombia)*	*Transformaciones urbanas, participación comunitaria y salud: aprendizajes de Brazil, Chile, y Colombia* (Urban transformations, community participation, and health: Lessons from	UniAndes, Colombia<br>PUC<br>Federal University of Minas Gerais<br>Local policymakers and community members from each locality	Spanish, Portuguese

	Brazil, Chile, and Colombia)		
28 September 2021<br>*Virtual (Brazil)*	*Mobilidade, Poluição Do Ar E Mudanças Climáticas: Desafios Para As Cidades Da América Latina* (Mobility, Air Pollution and Climate Change: Challenges for Latin American Cities)	University of São Paulo<br>Federal University of Minas Gerais<br>CDP (the Carbon Disclosure Project)<br>Municipal Secretary of Transport and Mobility	Portuguese
28 October 2021<br>*Virtual (Brazil)*	*Como os alimentos chegam e são distribuídos nas cidades?* (How does food move around our cities?)	The Oswaldo Cruz Foundation (FIOCRUZ)<br>Federal University of Minas Gerais<br>Local policymakers and community representatives	Portuguese
14 September 2022<br>*Virtual (Guatemala)*	SALURBAL Symposium: Promoting child health in urban environments	INCAP, SICA<br>Drexel University	Spanish
31 January 2023<br>*Virtual (Brazil, USA)*	Making the invisible, visible: Race, racism, and health data	The Ubuntu Center on Racism, Global Movements and Population Health Equity<br>Drexel University Urban Health Collaborative<br>CIDACS<br>Iyaleta<br>Pan-Diaspora Project<br>McGill University	Portuguese, Spanish, English
March 2023<br>*Mexico City, Mexico*	SALURBAL Project results and their relevance for the Mexican context	National Institute of Public Heath, Mexico (INSP)<br>Pan American Health Organization<br>Metropolitan Area Air Quality Program<br>Secretary for Health Promotion	Spanish

Supplementary Table 3: SALURBAL's locally focused, in-person and virtual dissemination events, including date, theme or topic, a list of host and partner institutions, and language are noted.

Supplementary Table 4

**Indicator**	**Description**	**2017-2023**
Unique website page visits	Total number of unique page views, excluding repeat visits	83,391
Website visitor countries	Number of countries from which the project's website has been accessed	170
Newsletter subscribers	The number of individuals signed-up to receive the project's quarterly digital newsletter	479
Twitter followers	The number of total followers the project has on Twitter	1,736
Average monthly Twitter impressions	The average number of impressions Tweets from the project received per month throughout the project	16,000

Virtual dissemination events	Number of virtual events hosted by SALURBAL (LAC-Urban Health webinar series, Diálogos SALURBAL, and other events) that aimed to disseminate findings to policy actors	13
YouTube views	The number of views all project videos and recordings (including virtual event recordings) have received	2,748
Data and policy briefs	The number of data and policy briefs produced by SALURBAL	12
Blog posts	The number of posts published on SALURBlog	60
Media releases	Total number of media releases created and shared with journalists	13

Supplementary table 4: Select digital dissemination and communications metrics for SALURBAL's website, social media, print materials, virtual events, and media engagement.
